# Supplementary material for: Clustering of home delivery in Bangladesh and its predictors: Evidence from the linked household and health facility level survey data
Source: PLOS Glob Public Health. 2024 Feb 15;4(2):e0002607. doi: 10.1371/journal.pgph.0002607 (PMC10868736; doi:10.1371/journal.pgph.0002607)
Supplement: Attachment — Submitted filename: renamed_3298a.docx [file pgph.0002607.s001.docx]

**Authors’ responses to reviewers’ comments**

| **Reviewers’ comment** | **Authors’ response** |
| --- | --- |
| **Reviewer#1** |  |
| Thank you very much for the opportunity to review this well-written and interesting manuscript. The authors tackle an important question – predictors of home birth in Bangladesh – using a novel approach. They make an important contribution to the literature by showing substantial geographic variation in home births and identifying important predictors of home birth.  Please see my comments below. | Thank you for your useful comments. I have addressed each of your comments and provided a point by point response below. |
| **Methods:** |  |
| 2) It would be very helpful if you could include clearer definitions of “hot spots” and “cold spots.” | We have included definitions of hot spots and cold spots in the abstract and in the manuscript at their initial mention. In the abstract, the pertinent sentence reads:  *Moran's I was used to determine hot spots (geographic locations with notably high rates of home delivery) and cold spots (geographic areas exhibiting significantly low rates of home delivery) of home delivery.*  In the main body of the manuscript, the relevant sentences in the results section read-  *The Getis-Ord Gi* statistic indicated significant clustering (p < 0.01) (Fig. 1). Cluster-level spatial analysis (Figure 1) identified significant hot spots (geographic locations with notably high rates of home delivery) of non-supervised and unskilled supervised home delivery in Dhaka, Khulna division, parts of Rajshahi and Rangpur division, as well as areas in Barishal and Chattogram division. Conversely, significant cold spots (geographic areas exhibiting significantly low rates of home delivery) were observed in Sylhet and Mymensingh divisions, along with some parts of Barishal, Rangpur, and Rajshahi divisions.* |
| 3) In addition, I think the current use of the term “clusters” is somewhat confusing. I think you might be using it in two separate ways: the first usage is to define the DHS clusters (used for sampling), and the second is to define geographic groupings of home births (as in “prevalence of home birth clusters”). I would recommend clarifying the usage of this term throughout the manuscript. | We have now defined cluster in its first use in the study design and population section under the Methods. This now reads-  *Firstly, 675 clusters (an area with 100/120 households) were randomly chosen from a list of 293,579 clusters in Bangladesh, generated based on the 2011 Bangladesh National Population Census.* |
| **Results:** |  |
| 1) In the justification for using GWR over OLS, it would be useful to hear your reflections on whether and why GWR might be the more appropriate way to model these data (based on theory about the relationships being modelled, as opposed to R-squared and AIC). | We have added the following sentence in the statistical analysis section to address your comment.  *The reason for using GWR was to provide cluster-level estimates for explanatory variables concerning their effects on home delivery that other regression models, including ordinary least square (OLS) regression model, could not provide; they only offer the average estimate for the sample.* |
| 2) Figures 2-4 – I found the discussion of these results very interesting, but the figures somewhat hard to understand. It would be helpful if you made the legends larger. Additionally, I’m not sure I understand the coefficients. Why are ranges given instead of point estimates with confidence intervals? Is this because the coefficients are estimated separately within every DHS cluster? Finally, would it be possible to use colors in a way that aligns with the numerical values (e.g., one color for each panel, but different color densities to indicate higher vs lower coefficients)? | We have revised each figure legend, enlarging them for better clarity, addressing your comment. You correctly understood that we presented a range instead of point estimates since coefficients are estimated separately for each cluster. Unfortunately, your last point about using a single point is not feasible due to the varied range of coefficients. Please check the revised version of the manuscript. |
| 3) “In hotspot areas, partner illiteracy, having more children, and the partner's occupation as an agricultural worker were significant predictors. Conversely, in cold spot areas, these characteristics had less impact on reducing home delivery.”…Should this instead say, “Conversely, in cold spot areas, these characteristics had less impact on increasing home delivery.”? | We have edited the relevant sentence as per your comment. This now reads-  *…….Conversely, in cold spot areas, these characteristics had less impact on increasing home delivery……* |
| 4) “The study identified community-level illiteracy as a significant predictor of non-skilled personnel supervised home delivery.” – Was this true in both hot spots and cold spots? | We have edited the relevant sentence to make it clear. This now reads-  *The study identified community-level illiteracy as a significant predictor of non-skilled personnel supervised home delivery in hot spots areas.* |
| 5) In the results from the GWG model, the authors discuss results from hot spots and cold spots. Are they able to also comment on the regression results in the ‘insignificant’ spots, or is that not feasible with this method? | You correctly understood. This is not feasible to comment on regression results in the insignificant spots. |
| **Discussion:** |  |
| 1) The authors’ statement on the prevalence of nonskilled personal supervised home birth in LMICs (54.8 to 70.8) seems very high. For example, this recent study of DHS surveys says that 1 in 3 women in LMICs delivers at home "Prevalence of home birth among 880,345 women in 67 low- and middle-income countries: A meta-analysis of Demographic and Health Surveys" - PMC (nih.gov). What is driving the difference across these different estimates? | The estimate we reported in our study aligns with the national-level estimate. Therefore, the finding is accurate. However, in response to your observation, we have now edited the relevant sentences and included your citation from the paper you referred to. The revised sentences now read-  *The observed prevalence of non-skilled personnel-supervised home delivery is 53.18, aligning with the findings of another study in Bangladesh [19]. However, this rate is significantly higher than the average in LMICs, where a recent study reported that 30% of total births occur at home [20].* |
| 2) The authors provide an interesting analysis of the reasons for clustering of home births. In addition to the reasons discussed, I was interested by the finding that women aged 20-34 are less likely to have a home birth. I wonder if this is an indicator the health system is doing a good job of identifying women outside this age range (<20 and 35+) as higher-risk and successfully steering them to health facility-based childbirth. | Thank you for this crucial observation. We have now added the following sentences to the discussion section to incorporate this issue into the manuscript:  *The age of women is also an important factor to consider. Hospitalized deliveries are often prioritized for women of comparatively younger and older ages, considering their higher risk of facing pregnancy complications. Therefore, healthcare providers at the community level often recommend hospitalized delivery for them as compared to younger women [4, 22].* |
| 3) In the discussion about the role of longer birth intervals, the authors could also mention the fact that, when women wait longer between pregnancies, they no longer have a very young child at home in need of their constant care. This might make facility-based childbirth more feasible for the second child. | We have added the following sentence in the discussion section to address this comment-  *Furthermore, women with a longer duration between pregnancies do not have a very young child at home requiring constant care, making it easier for them to decide on hospitalized delivery.* |
| 4) “Our analysis focused on estimating the prevalence of home birth clusters, while existing studies provided average estimates for all clusters within specific divisions.” – Can you explain more about what this means and why the results are different? I think it would be helpful for many readers if you included a clear definition of “prevalence of home birth clusters” (vs hotspots) early in the paper. | We have added the following sentences to clarify the issues you identified, along with adding definitions of hot spots and cold spots to address another comment. The added sentences read:  *Our analysis focused on estimating the prevalence of home births across clusters to clearly identify cluster-level differences. However, existing studies provided average estimates for all clusters within specific divisions, often masking clusters with high levels of home births.* |
| **Reviewer#2** |  |
| The authors present an interesting article on a topic of growing importance. With the limited published literature on this specific topic within the LMICs, their analysis is a valuable addition to the field.  I have few comments: | Many thanks for meticulously reading our paper. We have addressed each of your comments and provided a point-by-point response. Please see our response below. |
| 1. Under the Explanatory variables: 'The calculation procedure for this distance variable is available elsewhere [17]' While it is okay to indicate the references of some procedures in other papers, like this distance variable calculation, a summary of the methodology/procedure should be highlighted in the paper. please take note in all the references like such in the paper. | We have added the following sentences in the explanatory variable section of the manuscript to address this comment-  *We calculated distance in two stages. During the initial phase, we identified clusters of households where women reside in close proximity to health facilities offering hospital delivery care services. In the subsequent step, we utilized road communication system data from Bangladesh to calculate the on-road distance that later incorporated in the analysis. The calculation procedure for this distance variable is available elsewhere [17].* |
| 2. In table 1. under Preceding birth interval. Please check '2 years' and specify which group it belong. it is apparently in the two groups. | Many thanks to bring this typo in our attention. We have now addressed it. |
| 3. Be consistent in your decimals. some p values are in 2 decimals and some in 3 decimals. Please be consistent across the paper. | We have now checked this issue throughout the manuscript and corrected it. |
| 4. There are some minor edits in the text needed to be done. | We have thoroughly reviewed the manuscript and edited the English where necessary. |
